# Application of Machine Learning to Study the Association between Environmental Factors and COVID-19 Cases in Mississippi, USA

**DOI:** 10.3390/math10060850

**Published:** 2022-03-08

**Authors:** Francis Tuluri, Reddy Remata, Wilbur L. Walters, Paul. B. Tchounwou

**Affiliations:** 1 Department of Industrial Systems & Technology, Jackson State University, Jackson, MS 39217, USA; 2 Department of Atmospheric Sciences, Jackson State University, Jackson, MS 39217, USA; 3 College of Sciences, Engineering & Technology, Jackson State University, Jackson, MS 39217, USA; 4 Department of Biology, Jackson State University, Jackson, MS 39217, USA

**Keywords:** Python programming, machine learning, linear correlation, linear regression model, COVID-19

## Abstract

Because of the large-scale impact of COVID-19 on human health, several investigations are being conducted to understand the underlying mechanisms affecting the spread and transmission of the disease. The present study aimed to assess the effects of selected environmental factors such as temperature, humidity, dew point, wind speed, pressure, and precipitation on the daily increase in COVID-19 cases in Mississippi, USA, during the period from January 2020 to August 2021. A machine learning model was used to predict COVID-19 cases and implement preventive measures if necessary. A statistical analysis using Python programming showed that the humidity ranged from 56% to 78%, and COVID-19 cases increased from 634 to 3546. Negative correlations were found between temperature and COVID-19 incidence rate (−0.22) and between humidity and COVID-19 incidence rate (−0.15). The linear regression model showed the model linear coefficients to be 0.92 and −1.29, respectively, with the intercept being 55.64. For the test dataset, the R^2^ score was 0.053. The statistical analysis and machine learning show that there is no linear dependence of temperature and humidity with the COVID-19 incidence rate.

## 1. Introduction

The virus SARS-CoV-2 is a member of a large family of viruses called coronaviruses [1,2]. As the incidence of Coronavirus Disease 2019 (COVID-19) began to increase rapidly across the world [3], the World Health Organization (WHO) declared a global pandemic on 11 March 2020 [4].

Similar to the coronavirus family, COVID-19 is an infectious disease, and human-to-human contact is the primary factor of transmission of the virus–by touching infected surfaces and then mediating the infection through the mouth, nose, or eyes. The complexity and gravity of the situation also led machine learning investigators to understand the mechanism of the spread of the disease with a view to control and mitigate. Machine learning is a non-invasive tool that acts on a large dataset of observations to find association features among the data. Machine Learning is being used in different research fields and applications such as genetic programming for the nondestructive testing of critical aerospace systems [5], machine learning-based detection techniques for NDT in industrial manufacturing [6], and machine learning in medical imaging [7]. Similarly, machine learning can be applied to COVID-19 data to predict useful features from the complex data in contrast to using a traditional computation-based method. Particularly, machine learning with COVID-19 data can be used to deduce risk factors related to weather, air quality, social habits, demographics, and location. A recent surveys on applications of machine learning for the COVID-19 pandemic is provided by Kushwaha et al. [8]. Hybrid machine learning methods are also used to predict the time series of infected individuals and mortality rate [9]. Machine learning is also utilized to accurately predict the risk for critical COVID-19 [10]. Some machine learning methods are studied to compare their performance in terms of COVID-19 transmission forecasting [11].

Apart from using machine learning for the prediction of COVID-19 transmission, the scientific community has sought to study and understand the impact of environmental factors such as temperature and humidity on the prevalence of COVID-19.

The survivability and persistence of SARS-CoV-2 depend on weather conditions that indirectly control the virus transmission. The association between weather variables and COVID-19 transmission is complex. Some studies have shown that weather factors such as humidity have a determining factor for virus survival in aerosols [12,13]. The effect of sunshine on the transmission of pathogens is not positive [14]. Yasir et al. [15] showed that humidity was associated with a lower incidence of COVID-19, and lower death rate; whereas temperature was associated with higher daily incidence and death rate due to COVID-19. Colin et al. [16] pointed out that weather probably influences COVID-19, but not significantly compared to other preventive measures. Merow et al. [17] investigated the seasonality and uncertainty of global COVID-19 growth rates and reported that uncertainty remains high in establishing an association between them.

The study by Gupta et al. [18] on the effect of weather on COVID-19 spread showed that it is possible to predict vulnerable regions with high chances of weather-based spread in already affected countries, and countries with high populations, such as India. Zohair et.al [19] studied the association between weather data and COVID-19 to predict mortality rate using a machine learning approach.

Given the continued interest of the scientific community in the role of weather factors on COVID-19, there is a need to consider local prevailing cases and weather in order to identify an association between them, and to examine, on a local scale, if a rise in temperature or low humidity decrease the transmission of the disease and hence reduce the number of COVID-19 cases.

In the present study, we examined the effect of weather factors on COVID-19 cases in Jackson, MS, USA, to understand and predict its potential association with weather factors. We also seek to determine if local weather conditions could be a factor in the spread of COVID-19. Statistical and machine learning methods will be used to corroborate the results.

## 2. Materials and Methods

### 2.1. Data Sources

Daily cases of COVID-19 in MS, USA were obtained from the Department of Health, MS, USA [20] and the incidence rates were computed. The weather data used for the study included temperature, humidity, dew point, pressure, wind speed, and precipitation. Daily averages of the weather data were taken from Weather Underground [21] for the same region and the period of study. It was assumed that the weather conditions of the neighboring regions did not vary much from that of Jackson, MS, USA. The period from 22 January 2020 to 4 August 2021, was selected due to simultaneous weather and COVID-19 data availability. The Mississippi region was selected to identify local effects. The cumulative dataset consisted of daily COVID-19 incidence rates, temperature, humidity, dew point, pressure, wind speed, and precipitation. For a cross-correlation analysis, COVID-19 incidence rates were used. Table 1 shows a sample of the collected data.

Using statistical methods and a machine learning model, the data were analyzed to determine the correlations between weather factors and COVID-19 incidence rate, if any, and to make inferences that would help policymakers to take preventive measures.

### 2.2. Analytical Procedures

The Scikit-learn module of Python 3 [22–26] was used to analyze the data and identify a correlation between the weather data and COVID-19 incidence rate using machine learning. Here, it was assumed that high temperature and humidity would decrease the incidence of COVID-19 cases. In the present work, a linear-regression machine learning model was applied to the dataset to determine the relationship between weather-data variables and the spread of COVID-19 and to draw inferences, if any exist. The linear algorithm was selected to predict the COVID-19 incidence rate from its dependence on environmental factors. A Jupyter Notebook was used to run the Python code on the NVIDIA Xavier NX developer kit [27].

For each variable of the dataset, plots of the daily values were obtained. Exploratory data analysis (EDA) was conducted to determine the frequency, mean, standard deviation, minimum, maximum, and quantiles. To understand the inter-relationships between the variables of the data, a cross-correlation analysis was carried out.

### 2.3. Machine Learning Model

In addition to the cross-correlation analysis, a linear-regression machine learning model [19,22–26] was run to determine model fitting for the relationship between the COVID-19 incidence rate and the humidity and temperature taken from the weather data. See Table 1 for the features used to train the linear model. Here, the input features (X) of the model are limited to humidity and temperature because of the assumption that high temperature and humidity would decrease the spread of COVID-19 cases. The target variable (Y) of the linear model is the COVID-19 incidence rate. The methodology of linear models for implementation in Python is well documented [22,26]. The general form of the linear model [22,26] is given by,

(1) Y=B0+B1×X1+B2×X2,

where Y is for the COVID-19 incidence rate, X1 is for humidity, and X2 is for temperature. The corresponding model coefficients are represented by B1, and B2, respectively, with B0 being the coefficient for the intercept.

The dataset consisting of weather data and the COVID-19 incidence rate were divided into two parts, namely the training data set and the testing data set. The model training was run on the training data set, and the test set which was not included earlier was used for validation and prediction. The performance of the model was evaluated by standard performance evaluation metrics, namely R^2^ (R-square metric), Mean Absolute Error (MAE), Mean Squared Error (MSE), and Root Mean Square Error (RMSE).

## 3. Results

### 3.1. Time Series Analysis Results

**Figure 1.**
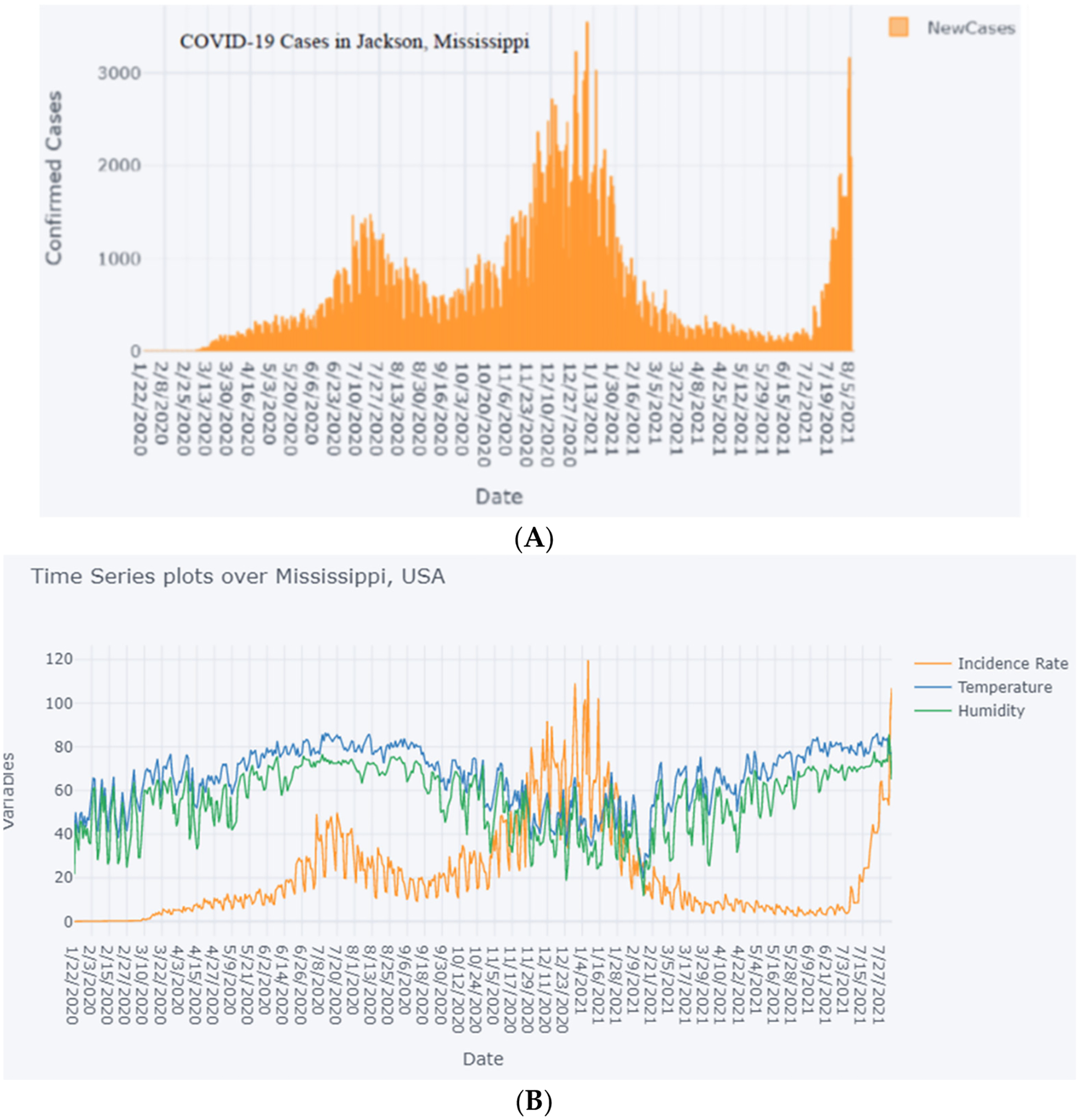
(**A**) Time series of COVID-19 cases (new incidence) for Mississippi 22 January 2020 to 4 August 2021. (**B**) Time series of COVID-19 incidence rate, temperature, and humidity for Mississippi 22 January 2020 to 4 August 2021.

A sample of the time series of COVID-19 cases, temperature, and humidity over, Mississippi for the period of study 22 January 2020 to 4 August 2021, is shown in Figure 1A,B.

### 3.2. Exploratory Data Analysis Results

The results of the EDA analysis are shown in Table 2. The mean number of COVID-19 cases was 633 (with a minimum = 0, and maximum = 3546) during the period of study in Mississippi. For each of the variables, the mean, minimum, and maximum values are as follows: Temperature: 65.9 °F, 19.6 °F, and 86.4 °F, respectively; Humidity: 55.8%, 11.6%, and 85.7%, respectively; Dew Point: 72.5 °F, 40. 5 °F, and 93.5 °F, respectively; Wind Speed: 6.42 mph, 0. 5 mph, and 17.2 mph, respectively; Pressure: 29.7 Hg, 29.2 Hg, and 30.2 Hg, respectively; Precipitation: 0.17 in, 0, and 3.6 in, respectively; and COCVI-19 incidence rate: 21.43, 0, and 119.7, respectively.

### 3.3. Cross-Correlation Analysis Results

**Figure 2.**
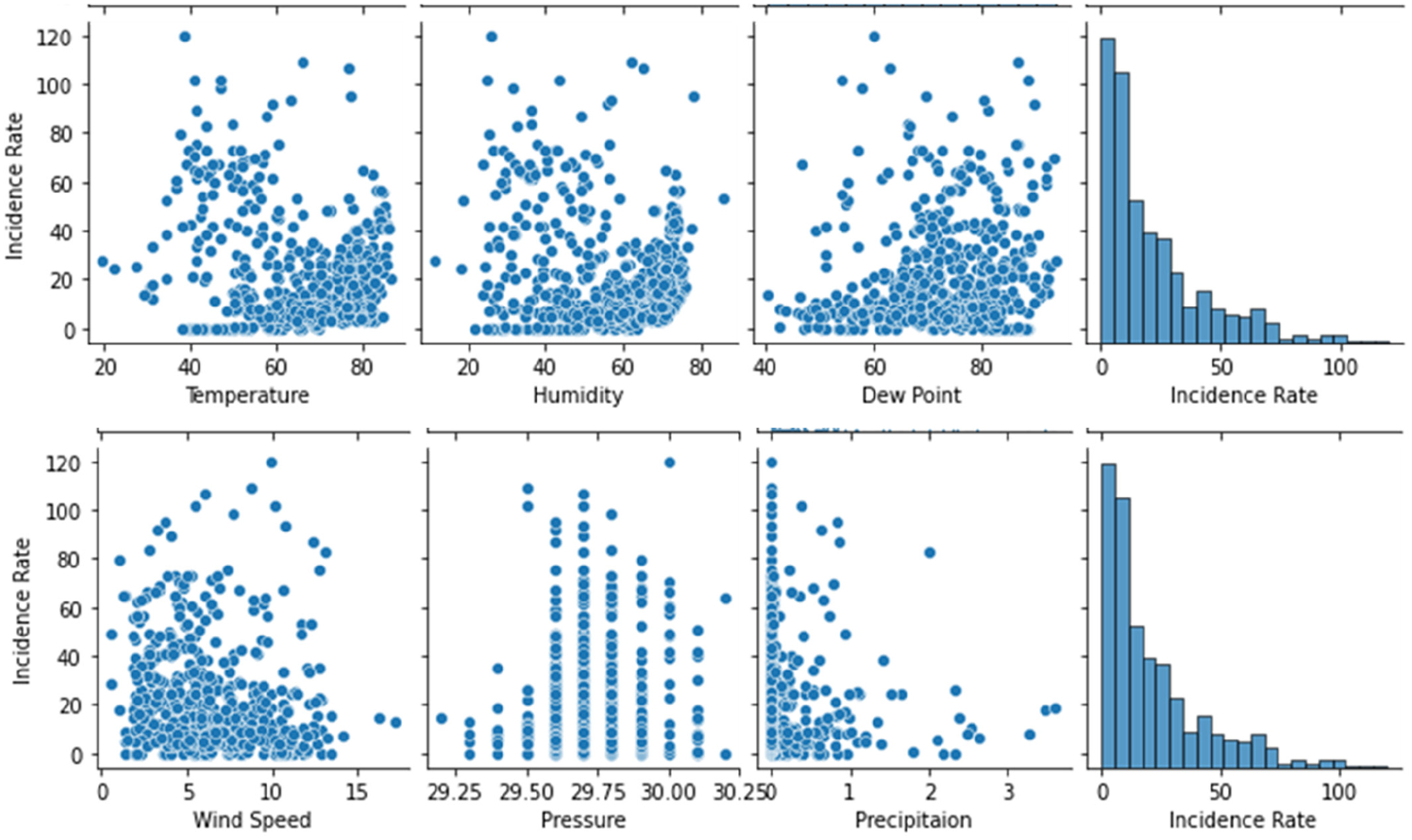
A scatter plot of COVID-19 incidence rate against each of the weather data variables (Temperature, Humidity, Dew Point, Wind speed, Pressure, and Precipitation) in Mississippi 22 January 2020 to 4 August 2021.

The results of the cross-correlation analysis are shown in Table 3. A scatter plot of the COVID-19 incidence rate against each of the weather data variables (Temperature, Humidity, Dew Point, Windspeed, Pressure, and Precipitation) is shown in Figure 2.

The correlation coefficients between the COVID-19 incidence rate and the weather variables (Temperature, Humidity, Dew Point, Wind Speed, Pressure, Precipitation) are −0.221, −0.148, 0.143, −0.155, 0.089, and −0.049, respectively.

**Figure 3.**
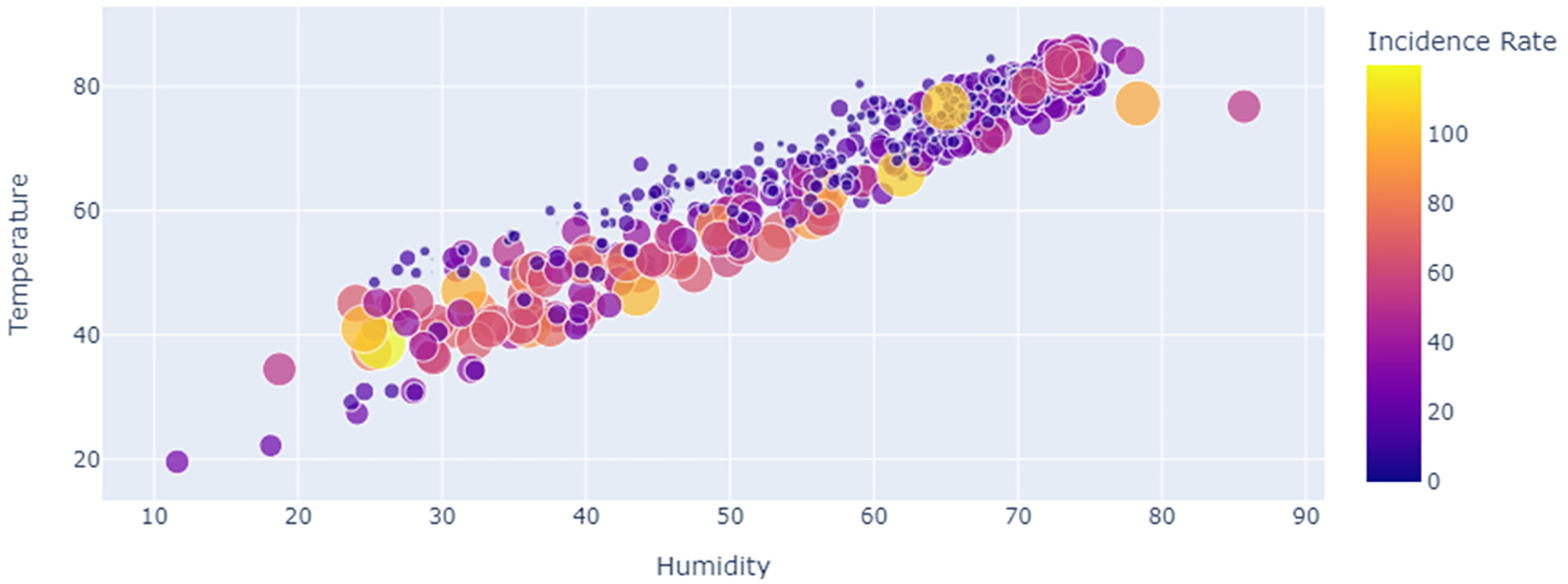
Correlation between humidity and COVID-19 cases in Mississippi, as a function of temperature 22 January 2020 to 4 August 2021.

Figure 3 shows the correlation between humidity and COVID-19 cases in Jackson, MS, USA, as a function of temperature for the period of study 22 January 2020 to 4 August 2021.

### 3.4. Machine Learning Model Results

**Figure 4.**
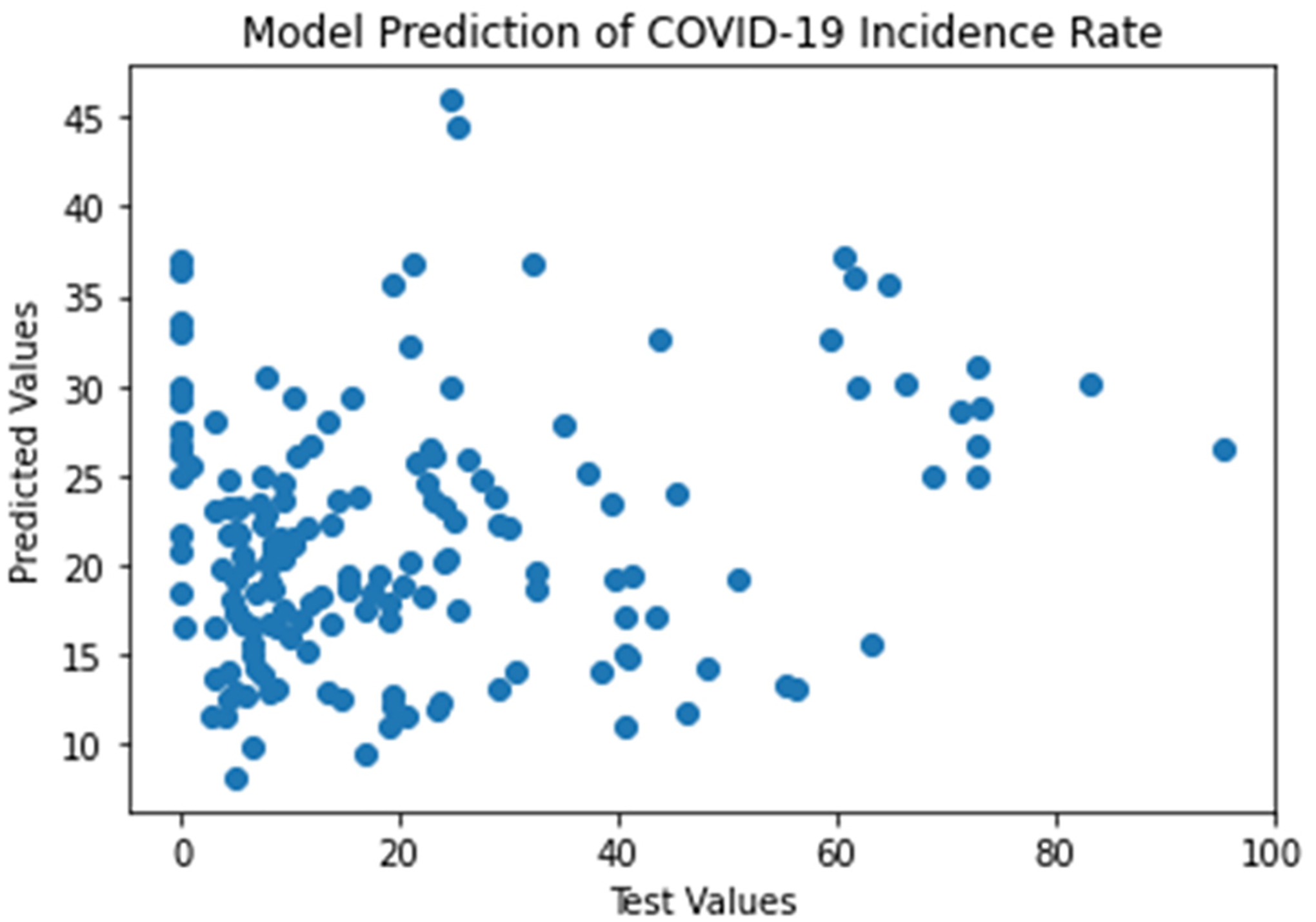
A scatter plot of test values vs. predicted values over Mississippi 22 January 2020 to 4 August 2021.

A linear regression machine learning model [22,26] was run on the data set. By applying Equation (1), the model coefficients were computed. The values of model coefficients B1, B2, and B0 are 0.92, −1.30, and 55.64, respectively. The model performance evaluation metric values of R^2^, MAE, MSE, and RMSE are 0.053, 15.25, 457.04, and 21.38, respectively. The linear model results are summarized in Table 4. A scatter plot of test values vs. predicted values over Mississippi 22 January 2020 to 4 August 2021 is shown in Figure 4.

## 4. Discussion

Among the six weather variables of the dataset of COVID-19 and weather data in Jackson for the period of study from 22 January 2020 to 4 August 2021, the statistical description of data (Table 3) shows a considerable variation in the range of values corresponding to temperature (from 19.6 °F to 86.4 °F), humidity (11.6% to 85.7%) and dew point (40.5 °F to 93.5 °F). However, the cross-correlation analysis (Table 3, Figures 2 and 3) shows either a slight positive or negative correlation of the COVID-19 incidence rate with these weather data variables, of −0.221, −0.148, and 0.143, respectively. Regardless, we carried out a linear regression model to run these variables so as to test the hypothesis that an increased temperature and humidity would decrease the spread of COVID-19 cases. The results of the linear regression model shown in Table 4 and Figure 4 show that the R^2^ value of 0.0529 is too small to consider any linear dependency between COVID-19 and the input features of temperature and humidity. The results of the machine learning model also agree with that of the results of the statistical method (Figures 1B and 3). The results of the statistical method do show a linear dependency between temperature and humidity but not with COVID-19 incidence.

There is an increasing interest in understanding the regional effects of weather factors on COVID-19 to reduce the large-scale impact of COVID-19 on mortality or health disorders. More specifically, identifying incidence rates and distribution in semi-rural and rural plain geographical terrain with relatively poor populations is not addressed. It is a common understanding that a rise in temperature or low humidity will decrease the transmission of the disease and hence reduce the number of COVID-19 cases. Our results also agree with the findings described by Colin et al. [16] that weather probably influences COVID-19, but not significantly compared to other preventive measures, and by Merow et al. [17] that uncertainty remains high in establishing an association between seasonality and COVID-19 growth rates. However, the present study provides a relatively efficient method of studying weather impacts on the COVID-19 incidence rate that would be useful for policymakers in terms of taking preventive measures.

## 5. Conclusions

This study illustrates that the association between weather variables and the COVID-19 incidence rate is not statistically significant in the study region. The computed values of correlation coefficients were −0.221, −0.148, 0.143, −0.155, 0.089, and −0.049 between the COVID-19 incidence rate and temperature, humidity, dew point, wind speed, pressure, and precipitation, respectively. Additionally, a low R^2^ score of 0.053 was generated from the machine learning model, rejecting the hypothesis that increased temperature and humidity would decrease the spread of COVID-19 cases in the study region.

## Figures and Tables

**Table 1.** A sample of data set collected—COVID-19 incidence rate and weather data over Mississippi 22 January 2020 to 4 August 2021.

Date	Temperature °F	Humidity %	Dew Point °F	Wind Speed mph	Pressure Hg	Precipitation in	Incidence Rate
1/22/2020	39.4	21.7	50.5	6.1	29.9	0	0.00
1/23/2020	50	44.8	82.8	7.3	29.6	0.02	0.00
1/24/2020	43.7	37.9	81.5	6.3	29.7	0.59	0.00
1/25/2020	40.6	32.5	75.7	1.4	29.8	0	0.00
1/26/2020	49	45.7	88.3	3.8	29.7	0.03	0.00
7/31/2021	83.1	74.3	73.4	2.1	29.7	0	56.16
8/1/2021	84	73	74.1	4.5	29.7	0	56.23
8/2/2021	76.8	85.7	72.2	5	29.7	0.02	53.15
8/3/2021	77.3	78.3	69.5	3.7	29.6	0.83	95.26
8/4/2021	76.8	65	63	6	29.7	0	106.85

**Table 2.** Exploratory data analysis of the data sets, including weather data and the COVID-19 incidence rate in Mississippi 22 January 2020 to 4 August 2021.

	Temperature °F	Humidity %	Dew Point °F	Wind Speed mph	Pressure Hg	Precipitation in	Incidence Rate
count	561.00	561.00	561.00	561.00	561.00	549.00	561.00
mean	65.88	55.84	72.50	6.42	29.72	0.17	21.43
std	13.67	14.61	10.73	3.01	0.15	0.46	21.91
min	19.60	11.60	40.50	0.50	29.20	0.00	0.00
25%	56.00	45.00	65.70	4.20	29.60	0.00	5.91
50%	68.30	59.10	73.40	6.10	29.70	0.00	13.34
75%	77.30	68.50	80.80	8.50	29.80	0.06	29.62
max	86.40	85.70	93.50	17.20	30.20	3.61	119.75

**Table 3.** Correlation between the variables of the data set. Variables include Temperature, Humidity, Dew Point,Wind Speed, Pressure, Precipitation, and the COVID-19 incidence rate over Mississippi 22 January 2020 to 4 August 2021

	Temperature	Humidity	Dew Point	Wind Speed	Pressure	Precipitation	Incidence Rate
Temperature	1.000	0.944	0.079	−0.086	−0.442	−0.002	−0.222
Humidity	0.944	1.000	0.394	−0.041	−0.551	0.083	−0.148
Dew Point	0.079	0.394	1.000	0.080	−0.448	0.262	0.143
Wind Speed	−0.086	−0.041	0.080	1.000	−0.184	0.198	−0.155
Pressure	−0.442	−0.551	−0.448	−0.184	1.000	−0.255	0.089
Precipitation	−0.002	0.083	0.262	0.198	−0.255	1.000	−0.049
Incidence Rate	−0.222	−0.148	0.143	−0.155	0.089	−0.049	1.000

**Table 4.** Linear Regression Model results.

Quantity	Value
Sample Size	556
B1; Humidity effect	0.92
B2; Temperature effect	−1.3
B0; Intercept	55.64
Mean absolute error	15.25
Mean squared error	457.04
RMSE	21.38
R^2^ score	0.053
